# The Paradoxes of Digital Tools in Hospitals: Qualitative Interview Study

**DOI:** 10.2196/56095

**Published:** 2024-07-15

**Authors:** Marie Wosny, Livia Maria Strasser, Janna Hastings

**Affiliations:** 1 School of Medicine University of St Gallen St.Gallen Switzerland; 2 Institute for Implementation Science in Health Care University of Zurich Zurich Switzerland

**Keywords:** health care, health care technology, health care information technology, hospital information technology, clinical information systems, health care professionals, experience, frustration, clinician burnout, technology implementation, paradoxes, digital tool, digital tools, hospital, hospitals, qualitative interview study, interview, interviews, Switzerland, thematic analysis

## Abstract

**Background:**

Digital tools are progressively reshaping the daily work of health care professionals (HCPs) in hospitals. While this transformation holds substantial promise, it leads to frustrating experiences, raising concerns about negative impacts on clinicians’ well-being.

**Objective:**

The goal of this study was to comprehensively explore the lived experiences of HCPs navigating digital tools throughout their daily routines.

**Methods:**

Qualitative in-depth interviews with 52 HCPs representing 24 medical specialties across 14 hospitals in Switzerland were performed.

**Results:**

Inductive thematic analysis revealed 4 main themes: digital tool use, workflow and processes, HCPs’ experience of care delivery, and digital transformation and management of change. Within these themes, 6 intriguing paradoxes emerged, and we hypothesized that these paradoxes might partly explain the persistence of the challenges facing hospital digitalization: the promise of efficiency and the reality of inefficiency, the shift from face to face to interface, juggling frustration and dedication, the illusion of information access and trust, the complexity and intersection of workflows and care paths, and the opportunities and challenges of shadow IT.

**Conclusions:**

Our study highlights the central importance of acknowledging and considering the experiences of HCPs to support the transformation of health care technology and to avoid or mitigate any potential negative experiences that might arise from digitalization. The viewpoints of HCPs add relevant insights into long-standing informatics problems in health care and may suggest new strategies to follow when tackling future challenges.

## Introduction

### Background

The burden placed upon health care professionals (HCPs) in hospitals by the complexities of digital environments is a recognized and well-reported challenge of our time [1,2]. While the increasing use of health information technologies (HITs), such as electronic health records (EHRs), clinical information systems, and clinical decision support systems, can improve patient care [3,4], suboptimal design and ineffectual workflow integration can lead to frustration and reduced job satisfaction and may be associated with burnout among HCPs [1,5,6]. In tertiary care facilities, nearly 50% of physicians’ daily schedules involve computer-based responsibilities, with EHR management consuming about 40% of their time [7]. Moreover, EHR design is known to correlate with clinician burnout due to information overload and high-volume messages from inbox notifications [8]. Burnout is experienced by 48% of hospital-based physicians, partially attributed to growing computerization, as well as time constraints, chaotic work settings, and other stressors [9,10], while in severe instances, it may lead to depression, substance abuse, and even suicide [11]. Furthermore, inadequate IT may contribute to systemic inefficiencies, diminishing overall economic efficiency within hospitals [12-14]. This consequently impacts national health care systems, which are already facing shortages in health care personnel [15].

The challenges related to technology usability in hospitals have persisted over decades [16-18], despite the regularly repeated promises of its potential to alleviate burdens [19-21], streamline clinical processes, such as data collection and synthesis; simplify documentation tasks [22]; enhance patient care; and improve the overall efficiency of health care systems [23]. One possible factor driving the persistence of challenges is the pace of change of the digital health care landscape. The result of each innovation that aims to address an existing challenge might create another challenge; for example, increasing digitalization may lead to the navigation of extensive data sets, management of a diverse array of digital tools, and the need to address ethical and data privacy concerns [2,24]. Furthermore, the growing use of unauthorized technologies, commonly referred to as *shadow IT*, is becoming more prevalent in hospitals, further complicating the digital health care ecosystem [25].

In the past, various theories have been developed to explain the factors influencing the adoption of technological innovations by individuals, such as the diffusion of innovation theory [26]; the Technology Acceptance Model that focuses on individuals’ decision-making processes regarding the adoption and use of technology [27]; and the Work System Model, which focuses on understanding and optimizing the interaction between people, processes, and technology within organizational work systems [28]. The application of these theories and models in health care technologies has been extensively studied, emphasizing the importance of considering organizational and professional culture, while also highlighting that human factors outweigh technological factors in health informatics [29,30].

In previous research, we conducted a systematic review of the published qualitative literature on HCPs’ experiences using digital tools in hospitals, with the aim to collect evidence on their benefits and challenges, particularly focusing on their personal experiences [31]. In this context, our findings revealed that clinicians’ personal experiences were reported less frequently compared with the moderators or outcomes associated with the use of HIT and that these can often not be distinguished. To delineate this distinction, we developed a theoretical framework that aimed to understand the complex interplay between the use of digital tools, encompassing positive and negative experiences, positive and negative moderators that possibly affect their adoption and use, and the resulting corresponding positive and negative effects and outcomes [31]. While outcomes of digital tools use in health care can encompass improved patient care, enhanced workflow efficiency, and increased information availability, moderators (eg, training quality and interface design) do exist, which can exert either positive or negative influences on tool use. Furthermore, individual user experiences, comprising personal thoughts, emotions, and feelings, can be sharpened by both tool outcomes and use, consequently either fostering or impeding the adoption of digital tools. These direct emotional experiences when using technologies may account for the downstream impact on well-being [31].

### Objective

Moreover, we discovered that the perspectives of HCPs themselves are often not sufficiently considered when designing technological solutions, despite evidence underscoring their importance in successful adoption [31-34]. To address this gap, this study aimed to gain new insights into the long-standing challenge of HIT from the perspective of HCPs through in-depth interviews by addressing the central research question “What is the lived experience of HCPs when using digital tools in hospital settings?” Switzerland offers a valuable research environment for a comprehensive exploration of HCPs’ perspectives due to its diverse health care landscape [35]. Understanding and amplifying HCPs’ experiences is vital to generate new insights into the mechanisms and tensions underscoring the ongoing challenges; explore current dilemmas and opportunities; and suggest alternative strategies for the future development, adoption, implementation, and evaluation of these tools.

## Methods

### Study Design and Setting

This qualitative descriptive interview study explored the firsthand experiences of HCPs using digital tools within hospital settings. Semistructured interviews were conducted to gain a deep understanding of HCPs’ experiences. Unlike other research methods, qualitative interviews allow us to capture intricate aspects of individuals’ experiences, including their thoughts, emotions, opinions, and perspectives [36-38]. Switzerland was chosen as the study location partly due to the limited exploration of its diverse health care digitalization landscape [31]. Moreover, Switzerland’s health care system is decentralized; therefore, there are a variety of solutions implemented at different hospitals, offering a rich source of diverse experiences. The reporting of this study adheres to the COREQ (Consolidated Criteria for Reporting Qualitative Research) guidelines (Multimedia Appendix 1) [39-41].

### Ethical Considerations

This study obtained ethical research approval from the academic institution and was deemed out of scope for national ethics approval. In this study, all participants provided verbal consent directly before the interview was conducted and audio recorded. Participants were informed that their participation was voluntary and that they could withdraw from the study at any time.

### Participant Recruitment and Selection Process

To participate, HCPs needed to be employed in a Swiss hospital, with at least 6 months of digital tool experience and willingness to participate in a 30- to 60-minute interview in the German or English language. Students and non–hospital-based HCPs were excluded. For recruitment, convenience sampling was performed via web-based platforms, social media, clinic newsletters, hospital intranet, and printed flyers. Moreover, the snowballing technique was used to further enhance diversity as well as purposeful sampling to cover various medical disciplines. The study’s purpose was communicated through the institute’s website and sign-up form. Six participants withdrew due to time constraints, suitability reevaluation, or unavailability.

### Data Collection Process

The authors conducted the interviews in person at hospitals, cafeterias, cafes, and the university or via video calls based on the participants’ availability. The interviews took place from May to August 2023 and were conducted until data saturation was reached, that is, when no new concepts emerged [42]. A semistructured interview guide was developed with the objective of understanding the HCPs’ experiences with digital tools. The questions were informed by the Technology Acceptance Model, which was used to understand and explain how HCPs perceive and adopt new technologies; the Work System Model, in order to explore how people, processes, and technology interact within a working system and to identify the different components of the health care work system; and a theoretical framework derived from our own previous systematic literature review, which explains the interplay between digital tool use, experience, moderators, and identified outcomes [31] (Multimedia Appendix 2) [27,28]. Interviews were conducted in the preferred languages of the HCPs—in Swiss German, German, or English. The interviews began with personal introductions and an outline of the study’s structure. Sociodemographic questions were followed by queries about digital tool experiences, including type, integration, usefulness, trustworthiness, professional image impact, collaboration, patient interactions, and reflections. In the end, participants were invited to ask questions or add thoughts on their digital tool experience that were not covered. Interviews were audio recorded and transcribed verbatim using the artificial intelligence (AI)–based software *Spoke* (Spoke Software, Inc) [43]. Transcripts were reviewed for accuracy, deidentified, and assigned unique study IDs.. Participants reviewed the transcripts, and revisions were made accordingly based on their provided feedback. In cases of no feedback within a month, the transcript was considered accepted.

### Data Analysis

Data collection and analysis proceeded concurrently by using ATLAS.ti software (ATLAS.ti Scientific Software Development GmbH) [40]. Deductive thematic analysis was used for the creation of a preliminary codebook based on the theoretical frameworks guiding the interview questions, followed by an inductive analysis to describe and synthesize HCPs’ experience of digital tools in hospitals [27,28,31,44,45]. Two authors independently familiarized themselves with the data by rereading the interview transcripts and applied codes to the transcripts in their original language. Through iterative discussions, codes were expanded, harmonized, and merged into a single codebook. Notes were taken to capture data items, patterns, and connections, forming preliminary themes, patterned responses, or meanings within the data [41]. The third author reviewed the transcripts to ensure comprehensive data coverage. As the next step, all 3 authors collectively examined initial codes and data extracts, inductively grouping them into themes specific to HCPs’ digital tool experiences in hospital settings. Themes were developed through analysis, combination, comparison, and visual mapping of code interrelations. Further examination led to the restructuring of themes and coded data into aggregated dimensions, ensuring a meaningful fit of codes and investigation of their alignment with the entire data set. Cross-connections among dimensions, themes, and subthemes were established. Narrative descriptions and definitions were created within the context of research inquiries into HCPs’ digital tool experiences, with the identification of emergent subthemes. Finally, the discussion of findings and analysis of codes on the meta-level culminated in the identification and definition of paradoxical experiences and perceptions among HCPs regarding digital tools in the hospital, depicted through opposing codes. Drawing upon Parse’s notion of paradox, a living paradox is delineated as a rhythmic oscillation of perspectives, with awareness arising from encountering the contradiction of opposites in daily navigation of value priorities while progressing toward the yet-to-be-realized [46]. To facilitate reporting, all quotes were translated into English. Finally, dimensions, themes, and subthemes were discussed with study participants to validate and ensure trustworthiness.

## Results

### Characteristics of Study Participants

A total of 52 interviews with an average duration of 27.38 (SD 9.1) minutes were conducted with HCPs representing 24 different medical specialties across 14 hospitals in Switzerland (Table 1). Sex distribution among the participants was balanced, with an average age of 40.31 (SD 10.18) years. Physicians comprised 71% (n=37) of the sample, with 42% (n=22) of the participants in senior roles and 29% (n=15) resident physicians. Registered nurses accounted for 29% (n=15) of the sample, with 19% (n=10) of the participants in senior positions and 10% (n=5) regular staff nurses. More than half of the HCPs (n=32, 62%) reported over a decade of experience with digital tools, typically acquired since the onset of their careers (Table 2).

**Table 1:** Distribution of study participants by medical disciplines (n=52).

Medical discipline	Participants, n (%)
Internal medicine	8 (15)
Neurosurgery	5 (10)
Emergency medicine	4 (8)
Intensive care	4 (8)
Oncology and hematology	4 (8)
Cardiology	3 (6)
Surgery	3 (6)
Transdisciplinary	3 (6)
Gastroenterology	2 (4)
Pneumology	2 (4)
Endocrinology and diabetology	1 (2)
Geriatrics	1 (2)
Gynecology	1 (2)
Hepatology	1 (2)
Infectious diseases	1 (2)
Nephrology	1 (2)
Ophthalmology	1 (2)
Orthopedics	1 (2)
Otorhinolaryngology	1 (2)
Pathology	1 (2)
Psychiatry	1 (2)
Radiation oncology	1 (2)
Radiology	1 (2)
Urology	1 (2)

**Table 2:** Sociodemographic and professional characteristics of study participants (n=52).

Participant characteristics			Participants
**Hospital size, n (%)**			
	Large (>700 beds, >7000 staff)	11 (21)	
	Medium (500-700 beds, 3000-7000 staff)	32 (62)	
	Small (<500 beds, <3000 staff)	9 (17)	
**Role, n (%)**			
	Senior physician	22 (42)	
	Resident physician	15 (29)	
	Senior registered nurse	10 (19)	
	Staff registered nurse	5 (10)	
**Sex, n (%)**			
	Male	27 (52)	
	Female	25 (48)	
**Age (y), mean (SD)**			40.31 (10.18)
	20-29, n (%)	10 (19)	
	30-39, n (%)	17 (33)	
	40-49, n (%)	14 (27)	
	50-59, n (%)	9 (17)	
	>60, n (%)	2 (4)	
**Experience with digital tools, n (%)**			
	6 months to 1 year	3 (6)	
	>1 year to 5 years	10 (19)	
	>5 years to 10 years	7 (13)	
	>10 years	32 (62)	
**Employment status (%), n (%)**			
	100	39 (75)	
	90	3 (6)	
	80	7 (13)	
	70	2 (4)	
	60	1 (2)	

### HCPs’ Experiences With Digital Tools in Hospitals

We identified 5962 quotations with 654 unique primary codes. Overall, negative sentiments about the use of digital tools have been mentioned more often by HCPs (n=3018, 50.62% quotations) compared with positive (n=1636, 27.44% quotations) or neutral (n=1308, 21.94% quotations) reports. Compared with our previous literature review and derived theoretical framework [31], which included 6 dimensions impacting digital tool use, including moderators, outcomes, and experiences, each of a positive and negative nature, this study reported a similar distribution of digital tool moderators, outcomes, and experiences (n=2976). Investigating these 6 dimensions, overall, 61.32% (1825/2976) of reported factors are moderators that influence the use of digital tools in positive (883/2976, 29.67%) or negative (942/2976, 31.65%) ways; 31.65% (942/2976) are outcomes, either positive (438/2976, 14.72%) or negative (504/2976, 16.93%); and only 7.02% (209/2976) true experiences and emotions, with only 1.88% (56/2976) positive and 5.14% (153/2976) negative reports that impact the overall well-being of clinicians.

**Figure 1:**
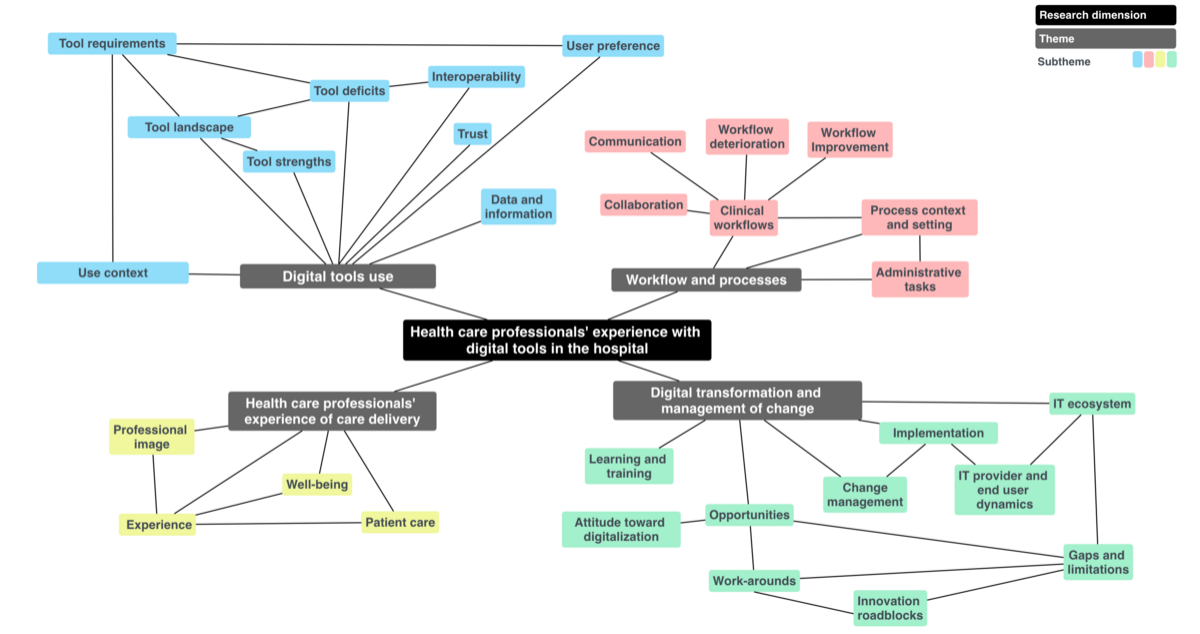
Thematic map analysis of themes and subthemes from qualitative interviews with health care professionals.

Four aggregated themes emerged from the thematic analysis of the interview data, namely, (1) digital tool use, (2) workflow and processes, (3) HCP’s experience of care delivery, and (4) digital transformation and management of change. Each theme in turn consisted of several subthemes (Figure 1).

### Theme 1: Digital Tool Use

HCPs (n=52) reported a comprehensive spectrum of >100 hardware and software tools, designed to serve both medical and administrative needs (Table 3). Reported tool strengths included remote access (n=15, 29%), system interoperability (n=14, 27%), and automation (n=11, 21%; Table 4 and Textbox 1). Reported tool challenges included interoperability issues (n=21, 40%) and technical malfunctions (n=21, 40%), compounded by counterintuitive (n=18, 35%) and user-unfriendly tool designs (n=17, 33%), as well as the existence of multiple individual tools (n=17, 33%) that sometimes ran in parallel and served identical purposes (Table S1 in Multimedia Appendix 3). These challenges lead to inefficiencies, tool avoidance, or the use of unauthorized shadow IT resources (n=12, 23%) such as private hardware, messaging apps, and social media platforms, which were often used for swift information sharing (Table S2 in Multimedia Appendix 3).

Participants (n=52) stated their preference for information systems that are both comprehensive and seamlessly integrated (n=18, 35%) and emphasized the importance of customization and modularity, aiming for a system that could be adapted to their specific needs, use contexts, and disciplines (n=6, 12%). For example, intensive care units and emergency medicine departments were reported to have distinct requirements, necessitating specialized tools that may not seamlessly integrate with the hospital’s broader systems (n=11, 21%). HCPs reported that they often found themselves in a position where tools did not align with their specific needs. However, they reported that they often had to adapt without the option to reject tools (n=20, 38%) or choose alternative solutions (n=11, 21%) because decision-making is orchestrated by management (n=10, 19%) with limited consideration for end users’ specific needs and preferences (n=12, 23%).

In the broader context, trust was central to digital tool adoption, and we observed an age-related difference (*P*=.02), with higher trust levels reported by HCPs aged 20 to 49 years compared to those aged ≥50 years. Nonetheless, most participants (n=36, 69%) expressed full trust in the tools, primarily due to oversight and safety confirmation offered by the local IT department (n=10, 19%).

**Table 3:** Most common reported digital tools in hospital settings (n=52).

Digital tools		Reports, n (%)
**Medical tools**		
	CIS^a^	52 (100)
	Dictation and speech recognition software	22 (42)
	Laboratory information systems	20 (38)
	Knowledge database	16 (31)
	Picture archiving and communication system	16 (31)
	Literature database	8 (15)
	Remote patient monitoring tool	8 (15)
	Surgery planning tool	8 (15)
	Digital imaging and communication system	7 (13)
	Surgical navigation system	6 (12)
**Administrative tools**		
	Email program	33 (63)
	MS Office 365 Suite	23 (44)
	Videoconferencing tools	16 (31)
	Shift scheduling software	12 (23)
	Calendar tool	9 (17)
	Performance tracking tool	9 (17)
	HR^b^ platform	6 (12)
	Administration tool	4 (8)
	Expense management tool	4 (8)
	Hospital bed management system	3 (6)

^a^CIS: clinical information system.

^b^HR: human resource.

**Table 4:** Selection of commonly reported subthemes of the 4 identified main themes (n=52).

Themes and subthemes			Participants, n (%)
**Digital tool strengths**			
	Remote access	15 (29)	
	System interoperability	14 (27)	
	Automation function	11 (21)	
	Provision of comprehensive overview	8 (15)	
	Intuitive tool design	7 (13)	
	Manual corrections possible	7 (13)	
	Automated data transfer	6 (12)	
**Digital tool deficits**			
	Interoperability issues	21 (40)	
	Technical malfunctions	21 (40)	
	Counterintuitive tool design	18 (35)	
	User-unfriendly tool design	17 (33)	
	Incompatible systems	17 (33)	
	Various specific and individual tools	17 (33)	
	Cumbersome tool visualization	13 (25)	
**Workflow improvement**			
	Gain in efficiency	37 (71)	
	Time-saving	34 (65)	
	Increased productivity	8 (15)	
	Better time resource management	6 (12)	
	Decreased workload	6 (12)	
	Mental support and assurance	4 (8)	
**Workflow deterioration**			
	Time consumption	34 (65)	
	Inefficiencies	22 (42)	
	More effort or cost than benefit	11 (21)	
	More distraction and less focus	9 (17)	
	No measurable outcome	9 (17)	
	Overflow of information	8 (15)	
	Work hindered	7 (13)	
**Positive experience**			
	Satisfied	14 (27)	
	Excited	8 (15)	
	Grateful	7 (13)	
	Relieved	7 (13)	
	Confident	5 (10)	
	Hope	5 (10)	
	Reassured	5 (10)	
**Negative experience**			
	Frustrated	46 (88)	
	Bothered and annoyed	21 (40)	
	Angry	12 (23)	
	Dissatisfied	12 (23)	
	Stressed	9 (17)	
	Helpless	8 (15)	
	Tedious	8 (15)

**Selection of supporting quotes on reported digital tool attributes, workflow impact, and user experience.**

**Digital tool strengths**

- “I’ll begin at home, where I have remote access. There, I can use the same interface as the one available at the hospital, and I find that very beneficial.” [Senior physician]
- “One of the aspects I truly appreciate about the clinical information system is that everything functions within a single program. You do not need to open any external applications.” [Resident physician]
- “What I found particularly advantageous was the seamless transfer of data from the surveillance monitor. It’s reassuring that you don’t have to painstakingly click through; it all happens automatically. That’s a huge relief.” [Staff nurse]

**Digital tool deficits**

- “I find it quite cumbersome because there are three different programs that don’t communicate with each other.” [Resident physician]
- “Moreover, the need to switch between these programs is inconvenient. They don’t integrate smoothly, and we’re certain that the key connection points between these systems are not 100% compatible. This often leads to issues.” [Staff nurse]
- “When a problem arises the initial answer is often, ‘We make a tool for that.’ Subsequently, someone, usually at a significant cost, begins developing a tool. However, it’s often rare to see truly effective, streamlined, and good results.” [Senior physician]

**Impact of digital tools on workflow improvement**

- “So, the patient curve generated electronically is identical to the one produced manually. That’s excellent.” [Resident physician]
- “It’s also a significant advantage that you can securely access information from different locations. For instance, in terms of planning, I can create the daily schedule from my office, while someone at the station office can simultaneously update that a patient is being discharged. I can instantly see these updates even when I’m not physically on-site.” [Senior nurse]
- “We have transitioned into a nearly paperless clinic. Our entire workflow has been digitized with the assistance of Care Paths defined within our IT infrastructure. Every aspect of our operations has been successfully integrated.” [Resident physician]
- “Working with this daily program can be quite frustrating due to its inherent inefficiency.” [Resident physician]
- “And then the phone rings, pulling you out of your workflow once again. You’re compelled to perform specific tasks on your phone, and only after completing those tasks can you return to your initial workflow. I believe this is a significant source of errors.” [Staff nurse]
- “However, I strongly believe that one’s efficiency increases when interruptions are minimized. [...] I’ve noticed that about half of the team, [...], tends to look at their phones or engage in other activities because they have received a message. This affects our interpersonal interactions, which are already rare. [...] I’ve observed that questions are often repeated, and important points get lost because some colleagues are distracted by notifications, messages, or emails [...]. Consequently, our morning meetings can easily become extended due to these digital distractions.” [Senior physician]

**Positive experiences of digital tool use**

- “It’s quite satisfying to swiftly access the data that’s relevant to you, enabling you to perform assessments and make necessary adjustments to therapies or conduct consultations.” [Resident physician]
- “I was able to schedule the session on my laptop, and it was incredibly satisfying. While it didn’t reduce stress, it certainly provided a sense of relief.” [Senior nurse]
- “The emotions involved were a mix of relief, in terms of time-saving, especially at midnight. Additionally, there was a sense of pride because this system is our creation, stemming from our team’s effort.” [Senior physician]

**Negative experiences of digital tools use**

- “You get completely blocked. I couldn’t access the clinical information system and I couldn’t work. It’s extremely frustrating.” [Staff nurse]
- “And then we have to cut back on patients. Or the resident physicians end up working overtime. When they have to deal with those interface management issues, they can’t be with the patients or leave early. That’s frustrating.” [Senior physician]
- “Undoubtedly, this places a heavy psychological burden on us because we’re aware that when the system isn’t functioning, we can’t take care of the patients who are in unstable conditions as they arrive. We must wait until the system is functioning again.” [Staff nurse]

### Theme 2: Workflow and Processes

While it was highlighted by the HCPs (n=52) that digital tools were promoting collaboration (n=24, 46%) and enhancing overall communication efficiency (n=7, 13%), the need for in-person interactions remained (n=18, 35%). However, it was reported that face-to-face interactions decreased (n=17, 33%) due to the use of digital tools and hospital logistics (n=5, 10%). Primary communication modes remained phone calls and emails, with some physicians having faced severe email overload (n=9, 17%) with >100 emails daily, while phone calls interrupted nurses’ workflows (n=3, 6%).

Components associated with workflow improvement attributed to digital tools were mainly seamless integration (n=22, 42%), supportive tool features (n=17, 33%), and correctly mapped care paths (n=10, 19%). These factors were described as enhanced operational efficiency (n=37, 71%), time management (n=34, 65%), and overall productivity (n=8, 15%; Table 4; Textbox 1). By contrast, HCPs disclosed that workflow challenges often stemmed from the coexistence of paper and electronic documents (n=23, 44%), leading to documentation errors, time inefficiency, and increased work effort (Table S3 in Multimedia Appendix 3).

In addition, HCPs (n=29, 56%) stressed that the time constraints and inefficiencies that they encountered were linked to the intricacies of administrative processes, along with bureaucratic impediments (n=11, 21%), which often were enforced through digital tools, underlined by the conflict and inherent complexity between balancing standardization and considering the individualization of medical procedures (n=17, 33%; Table S4 in Multimedia Appendix 3).

### Theme 3: HCPs’ Experience of Care Delivery

The integration of digital tools within the hospital ecosystem and HCPs’ (n=52) resulting experiences were multifaceted but primarily negative, characterized by frustration reported by most participants (n=46, 88%), annoyance (n=21, 40%), anger (n=12, 23%), dissatisfaction (n=12, 23%), and stress (n=9, 17%; Table 4 and Textbox 1). Notably, these negative experiences affect clinicians’ well-being, leading to work-life balance challenges (n=10, 19%), heightened stress levels (n=9, 17%), cognitive overload (n=7, 13%), and mental burden (n=5, 10%; Table S5 in Multimedia Appendix 3). By contrast, positive experiences associated with digital tools, although less frequently reported, include satisfaction (n=14, 27%), enthusiasm (n=8, 15%), gratitude (n=7, 13%), and relief (n=7, 13%), mainly arising from digital tools’ assistance with daily tasks (Table S6 in Multimedia Appendix 3).

The integration of digital tools significantly impacted HCPs’ professional image and self-perception, with half of the participants (n=28, 54%) experiencing shifts in their professional identity. This included positive aspects, such as feeling better informed (n=10, 19%) and more proficient (n=6, 12%). They often viewed themselves as forward-thinking, driven by a desire for enhanced digitalization in health care (n=19, 37%). Conversely, negative shifts in HCPs’ professional image were tied to increased time on computer tasks and documentation burdens (n=10, 19%), causing dissatisfaction due to role deviations and increased mental strain (n=5, 10%). Others (n=24, 46%) saw no significant changes, as digital tools were ingrained in their routines, particularly for those who grew up with digital tools (n=17, 33%).

Regarding decision-making, HCPs emphasized their irreplaceable human roles (n=8, 15%) based on their medical expertise and responsibilities. However, they also recognized significant reliance on digital tools (n=17, 33%) and their providers (n=3, 6%), especially in critical situations or system failures. Some HCPs noted changes in job expectations (n=5, 10%) and skillset requirements (n=5, 10%), including the need for IT proficiency, data interpretation, and rapid typing skills (n=5, 10%).

The core motivator for HCPs using innovative digital tools was to improve patient care and well-being (n=16, 31%), with an emphasis on patient safety (n=14, 27%), which was realized since HCPs were feeling better informed (n=10, 19%) and prepared (n=4, 8%) due to access to information. HCPs noted that digital tools had increased the quality of care (n=11, 21%), and mobile workstations facilitated improved patient-centered interactions (n=15, 29%). However, there were also reservations expressed about the potential transformation toward screen-centered care, which could lead to patients feeling disconnected and neglected (n=13, 25%).

HCPs anticipated the emergence of collaborative health care digital tools (n=6, 12%), emphasizing the importance of granting patients, their families, and caregivers access to health data for empowerment and shared decision-making (n=12, 23%), while also improving family and caregiver engagement through digital educational resources and visualization tools (n=2, 4%). Nevertheless, the abundant availability of medical information also raised concerns about misinformation and limited patient comprehension (n=2, 4%), particularly among patients with neurotic tendencies (n=3, 6%).

### Theme 4: Digital Transformation and Management of Change

The HCPs (n=52) stated that their digital proficiency relied significantly on effective training (n=21, 40%). Although initial in-person training sessions at job commencement were standard, they were often perceived as insufficient and generic (n=12, 23%; Table S7 in Multimedia Appendix 3). Specialized training (n=7, 13%) and learning from experienced colleagues in professional settings or informal interactions (n=27, 52%) were stated as more effective in addition to experiential learning (n=33, 63%) and trial-and-error approaches (n=13, 25%). Overall, it was noted that proficiency improved over time and practice (n=21, 40%). However, despite recognizing training’s importance, there was often a shortage of dedicated time (n=6, 12%) and a lack of prioritization (n=11, 21%), while controversially, an intriguing paradox emerged, with a subset of HCPs (n=4, 8%) reporting attendance in mandatory training that held no practical utility with their routines (Table S8 in Multimedia Appendix 3).

In the context of digital transformation, effective change management emerged as a pivotal factor for successful IT infrastructure implementation (n=9, 17%; Table S9 in Multimedia Appendix 3). Furthermore, HCPs reported that they perceived smaller organizations to be more agile, demonstrating greater success in IT implementation than their larger counterparts (n=9, 17%). While HCPs appreciated the iterative refinement of digital systems (n=8, 15%) and emphasized the significance of pilot projects with feedback mechanisms (n=7, 13%), others in contrast reported unsuccessful tool introductions (n=8, 15%) leading to subsequent discontinuations due to technical issues, high effort intensity, or nontailored processes with no measurable benefits (Table S10 in Multimedia Appendix 3).

It was noted that the health care industry lagged far behind in digitalization efforts compared to other industries due to its complex nature (n=17, 33%). HCPs expressed underutilized technical possibilities (n=28, 54%) and gaps, with better hospital systems (n=19, 37%) and superior tools that existed but were inaccessible in their organization (n=6, 12%). In particular, challenges with eHealth applications and the efforts toward a national Electronic Patient Dossier in Switzerland were reported as problematic, often with a sense of pessimism (n=8, 15%).

Numerous innovation roadblocks that lead to these gaps were described, including monetary constraints (n=15, 29%), regulatory hurdles (n=14, 27%), and organizational structures (n=7, 13%). In response, HCPs found work-arounds (n=22, 42%) and shortcuts (n=17, 33%), and some HCPs reported developing customized IT solutions (n=8, 15%). Nearly half of the HCPs (n=22, 42%) highlighted ongoing changes and new clinical information system implementation at their institutions, with some HCPs (n=11, 21%) actively involved in implementation efforts.

### Reported Paradoxes With Digital Tools in the Hospital

We identified 6 paradoxes associated with the experiences of HCPs with digital tools in the hospital (Table 5).

The first encompasses the “promise of efficiency and the reality of inefficiency,” which describes the striking contrast between anticipated efficiency gains from health care digitalization and the real-world challenges and inefficiencies experienced during and after implementation. Numerous HCPs conveyed that the digital technologies they then used were time-consuming, inefficient, and burdened with cumbersome processes, while others underscored the advantages in efficiency and time-saving measures.

The second paradox is the “shift from face to face to interface,” elaborating that health care team collaboration, spanning disciplines and extending externally, has transitioned from in person to digital interfaces, offering benefits and presenting challenges of maintaining a balance between both. HCPs noted that the transition toward screen-centric health care was reducing face-to-face interactions and had inadvertently hindered both internal and external collaboration along with regulatory constraints and systemic disparities. On the flip side, other HCPs have reported a prevailing reliance on in-person interactions as the default mode of operation. Moreover, it has been recognized that the integration of digital tools has enhanced communication efficiency.

The third paradox is defined as “juggling frustrations and dedication,” as HCPs experience daily frustrations with digital tools, which prove to be inefficient with a lack of user-friendliness and cumbersome interfaces, ultimately impeding workflows and causing frustration and contributing to emotional exhaustion. Furthermore, there have been reports of varying job expectations among HCPs, with some noting substantial shifts while others’ remained unchanged.

Another paradox is the “illusion of digital information access and trust,” describing the disconnect between information availability and practical accessibility. A large number of HCPs (47/52, 90%) expressed concerns regarding the quality of data and information, highlighting inconsistencies stemming from the existence of multiple and differing information sources and human errors during data replication. Moreover, it has been reported that limited access to information, coupled with challenges in locating relevant information, exacerbates inefficiencies and increases the likelihood of errors in medical decision-making. In contrast, some HCPs recognized the advantages of enhanced access to information, facilitating informed decision-making. Furthermore, a subset of HCPs noted improvements in data quality, indicating a positive trend in this regard.

Moreover, a paradox at “the intersection of clinician workflows and patient care paths” emerged, which emphasizes the complexities that arise in health care workflows due to insufficient health care IT, disruptions, computer-centric tasks, and nonstandardized care paths. Most HCPs (45/52, 87%) voiced concerns that current health care IT systems disrupted rather than streamline processes, leading to a deterioration in workflow. This degradation is attributed to the proliferation of digital technologies, which introduce challenges such as email overload and distractions, as well as the need to manage multiple tools simultaneously, often necessitating frequent switching between them. Nevertheless, amid these challenges, workflow improvements have been observed, such as fast access to patient data and informed decision-making, efficient documentation and communication, and resource optimization.

Finally, the last paradox describes the “opportunities and challenges of shadow IT” and how HCPs use unauthorized software and hardware to tailor solutions and address workflow needs and gaps efficiently while being aware of the potential risks and data privacy concerns. Some HCPs (12/52, 23%) disclosed their use of unauthorized tools such as generative AI, chat applications, and social media platforms to expedite communication and access to information, despite the inherent risk of sharing unauthorized patient data. However, this practice was frequently conducted discreetly, and at the same time, HCPs highlighted their awareness of the potential risks involved and emphasized the importance of prioritizing data privacy concerns.

**Table 5:** Identified paradoxes with opposing reports and supporting quotes.

Paradox and opposing reports (quotes, n)			Exemplary quotes
**The promise of efficiency and the reality of inefficiency**			
	Time consumption (n=79) Inefficiencies (n=52) Cumbersome processes (n=48)	“You have to click on the same things day by day...And it consumes so much time and is inefficient. We could instead dedicate that time to the patients instead of sitting tired in front of the computer...” [Junior nurse]	
	Gain in efficiency (n=122) Time-saving (n=68)	“I am actually a big fan of technologies...because they primarily make our daily lives significantly easier, make everything more efficient, and also make it safer.” [Assistant physician]	
**The shift from face-to face to interface**			
	Shift to screen-centric care (n=21) Reduced face-to-face interaction (n=32) Internal collaboration hindered (n=15) External collaboration hindered (n=15)	“I believe the direct interaction is gradually fading away. It feels like everyone is going their separate ways, immersed in their own tools, rather than functioning as a cohesive team.” [Senior physician]	
	In-person presence (n=22) Internal collaboration promoted (n=33) External collaboration promoted (n=18) Improved communication (n=9)	“We definitely have less face-to-face involvement with the nursing team. However...this is not necessarily negative, as nurses value having slightly less direct interaction...Now, with nurses able to quickly access information in the clinical information system...there are fewer disruptions in the processes. This...is beneficial, although we may have slightly less interaction as a result.” [Senior physician]	
**Juggling frustrations and dedication**			
	Negative emotions (n=197) Frustration (n=84)	“One feels incredibly hindered. I could not access my clinical information system, which prevents work. It is extremely frustrating.” [Junior nurse]	
	Positive emotions (n=71) Satisfied (n=27)	“The ultimate aim is for the diagnosis to automatically appear digitally for physicians, ensuring it remains on their radar for proper treatment...The more insight I have into the required care, the more effectively I can practice medicine.” [Senior physician]	
**The illusion of digital information access and trust**			
	Poor data quality (n=25) Different information sources (n=20) Limited access to information (n=19) Difficulties to find information (n=11)	“The quality of work has suffered due to this digitalization. Nowadays, diagnoses are simply copied from previous lists, and...are now six pages long because everything is copied and pasted.” [Senior physician]	
	More and better access to information (n=58) Improved data quality (n=7)	“On one hand, digitalization has certainly brought us forward. We now have more concrete data, and the quality of that data has significantly improved.” [Senior nurse]	
**The intersection of clinician workflows and patient care paths**			
	Workflow deterioration (n=407)	“Among colleagues, the tool is seen as a disruptor, rather than something helpful.” [Senior physician]	
	Workflow improvements (n=277)	“We have transitioned into a nearly paperless clinic. Our entire workflow has been digitized with the assistance of Care Paths defined within our IT infrastructure.” [Assistant physician]	
**The opportunities and challenges of shadow IT**			
	Use of shadow IT (n=29)	“Recently, I tried to use ChatGPT to write reports. I was really very satisfied because I can gain a lot of time even though it is not perfect.” [Assistant physician]	
	Data privacy importance (n=17) Data privacy concerns (n=8)	“Of course, security always entails some risk...I’m not really familiar with matters like data protection and such...But of course, it certainly also carries a risk.” [Senior physician]	

## Discussion

### Principal Findings

Despite the long-standing recognition of the potential of HIT, the realization of these promises seems perennially distant. Our study aimed to explore HCPs’ perspectives on the ongoing digital transformation in hospitals and unveiled numerous negative experiences and frustrations. Paradoxes emerged from HCPs’ perspectives, often highlighting opposing forces within reported experiences. Therefore, our findings underscore the substantial challenges posed by digital tools in hospitals and the impact of these challenges on HCPs’ well-being. The development and implementation of effective hospital IT systems and digital tools face a narrow and challenging path to navigate these paradoxes, which are represented by tensions that often push in opposite directions. Given this context and drawing from our findings, we discuss these emerging paradoxes as well as potential strategies that might help to navigate them.

### The Promise of Efficiency and the Reality of Inefficiency

One paradox that was reported is the apparent promise of technological innovation compared to the actual experience of HCPs. The desire for efficiency gains through streamlined processes and improved patient care has long driven the adoption of digital tools in hospitals, with the optimistic outlook that technology may revolutionize health care delivery and administrative workflows [47,48]. However, this study revealed significant disparities between the ideation (the imagined promise) and the actual results of using digital tools in real-world hospital settings. Motivated by the aim to align discipline-specific needs with standardized patient care, standardizing medicine presents a complex paradox in itself and creates an inherent tension between the need for unification and customization of clinical information systems [49]. The aim for hospital information systems to establish seamless and fully integrated systems compatible with diverse tools, workflows, data models, and legacy system integrations represents a serious challenge [50,51]. This is further compounded by the interdepartmental and intrahospital variations, stemming from discipline-specific and regional needs. In addition, hospitals must maintain operations during IT system transitions, which necessitates thorough preparation, weighing the benefits and drawbacks of a disruptive system replacement versus a phased approach [2]. Another essential factor for effective use and integration of technologies into clinical decision-making is adequate training [52], which is currently often neglected. Moreover, while promising claims, for example, about the accuracy of medical AI as a digitalization initiative suggest that it can boost efficiency, this overlooks crucial factors such as the narrow definition of workload and the significant role of human factors in technology implementation, where human knowledge, competencies, and trust are pivotal in determining efficiency outcomes [53]. Given that many of the inefficiencies and frustrations reported in our study were due to system malfunctions or access challenges, one strategy to mitigate the possibility of inefficiencies introduced by digital tools might be for hospital IT systems to offer multiple access points and the possibility of diverse interfaces around common data stores [54]. Modularity and redundancy would allow backups for information capture during tool unavailability or system downtime, as well as tailored interfaces for different stakeholders and workflows [55]. This would also enable a staged rollout of novel innovations, minimize the risk of widespread system failure, and align with the dynamic nature and introduction of health care technology innovations [56].

Another key aspect that was reported is the emerging shifts in HCPs’ identity, professional image, and self-perception due to digital technology integration. While some experience significant shifts, others, especially those accustomed to digital tools, see minimal to no changes. Nevertheless, providing tailored and effective training of these technologies and integrating these trainings into HCPs’ schedules or freeing up time are essential for harnessing the full potential of health care technology [57]. In this regard, effective training sessions could encompass immersive digital simulations, real-time feedback, personalized learning, and adaptive modules [58-60]. Moreover, knowledge-sharing mechanisms and a culture of continuous learning are crucial for maximizing digital system use [61] as well as interprofessional training sessions between disciplines [62].

### The Shift From Face to Face to Interface

Another paradox emerged in this study is the shift from traditional face-to-face interactions to digital interfaces, encompassing discipline-specific, interdisciplinary, multiprofessional, and external collaborations [63]. Multiprofessional collaborations in health care have been shown to improve patient outcomes while also benefiting HCPs by reducing redundant work [64]. However, this transition has not been without challenges, and there is a need for balancing in-person and digital collaboration, as HCPs noted distractions and a reduction in the ability to focus on human interactions during consultations since they increasingly rely on digital counterparts. Nevertheless, digital tools and increased data accessibility can promote all forms of collaboration through information sharing, remote access, and facilitation of swift consultations with colleagues. However, regulatory constraints based on regional mandates and the heterogeneous nature of the digital hospital systems currently limit external collaboration and set a major hurdle for a unified EHR approach. Achieving this requires harmonizing regulations and developing interoperable systems that enable the exchange of health information while upholding stringent data privacy standards and safeguards, especially in sensitive areas such as mental illness, substance use disorders, and sexual health, and ultimately ensuring adequate transparency while maintaining patient confidentiality [65,66]. To alleviate these challenges involved in the transition from face to face to interface and reduce digital overload, the introduction of communication guidelines and email etiquette is crucial for reducing stress, enhancing productivity, and fostering concentrated work phases. AI-supported systems can help to streamline these processes while enforcing a “no-devices” policy during time-boxed discussions, restricting email access, and optimizing inbox management, which can further increase engagement and minimize interruptions [67].

### Juggling Frustrations and Dedication

The multifaceted frustrations experienced by HCPs in their daily roles negatively impact their well-being. Frustrations caused by technology and the resulting inefficiencies in work processes are impeding HCPs in clinical and administrative tasks. These frustrations paired with a constant sense of availability are disrupting work-life balance and are linked to emotional exhaustion, which can lead to burnout [14]. In accordance with previous research, it was found that increased time spent with computer tasks due to administrative duties and bureaucratic procedures adds complexity to health care work, affecting patient care and HCPs’ well-being as well as career satisfaction [68]. One of the most striking findings of this study is the substantial frustration and stress that can emerge when digital tools do not function as expected, leading to delays in HCPs’ workflows and patient care. Concerns adding to this include not only tool reliability, security, and overall data privacy concerns but also missed opportunities, which all can lead to distrust or tool rejection. Moreover, the reported gap in access to necessary health care technologies and lack of decision rights further exacerbate HCPs’ frustrations. Comparing these findings with those of other studies confirms that the cumulative effect of current IT challenges coupled with staffing shortages can result in extended working hours, overwork, and frustrations and highlights the urgent need for solutions [69]. Our study emphasized the paradox and the need to reduce HCPs’ digital frustration while recognizing their job dedication. Involving HCPs in the choice, design, and implementation trade-offs in digital tool implementation is essential to empowering them as stakeholders and reducing associated negative emotions [70]. Moreover, harnessing the potential of innovations such as voice-based data entry and virtual smart assistants, as well modern language model technologies, can streamline administrative tasks, enhance data quality, and reduce costs [71].

### The Illusion of Digital Information Access and Trust

Another paradox is the enormous and increasing amount of available data and knowledge, which carries positive and negative implications for HCPs’ workflows related to information access, quality, and trust. Trust emerged as a central factor in the adoption of digital tools, with younger HCPs exhibiting higher levels of trust in digital tools compared to their counterparts aged ≥50 years. Research has consistently highlighted the significance of trust as a crucial component for the successful use and implementation of health care technologies. To establish trust, it is essential to align AI with existing values and consider social interactions and negotiations of values comprehensively [72].

One of the foremost obstacles reported is the illusion and disparity between information availability and actual accessibility, exacerbated by issues ranging from user account restrictions and unstable IT infrastructure to information overload and obscurity. The most critical issue reported is the duality of data quality. While robust data sets offer valuable insights and this is often a driver for adopting a digital strategy, HCPs frequently encountered data inconsistencies in patient reports due to various factors, including software limitations and human-generated copy-paste errors, which were also reported in other study settings [73,74]. This is especially critical for systems in which compromises in data integrity can lead to erroneous decisions affecting patient outcomes [75]. Hence, HCPs emphasized their role in making the ultimate medical decisions, underlining the need for expert knowledge to address data quality challenges. Moreover, limiting the copy-paste function in EHRs has been proposed to enhance report quality [76]; however, this would risk exacerbating the time spent in reporting. Participants in this study felt satisfaction when they had access to the right information at the right time, but in practice, what this meant differed from clinic to clinic and from HCP to HCP. To promote the desired flexible and individually customizable information systems to optimally support workflows within the realities of resource constraints, one possibility is to use the growing family of open-source infrastructures for dynamic, openly integrative solutions, which not only foster adaptability but also embrace collaboration [77].

### The Intersection of Clinician Workflows and Patient Care Paths

Moreover, our study highlighted the complexities and intersection of HCPs’ workflows and patient care paths, with most participants agreeing that they are increasingly exposed to a multitude of workflow interruptions and demands of administrative computer-centric work. Incessant email distractions have become a significant burden which, coupled with automated reminders and EHR alerts, create a constant challenge in prioritizing and managing correspondence. It has been indicated that information overload is raising physicians’ cognitive load and is negatively affecting patient safety [78], while the increase in computer-centric work, with some HCPs spending up to 80% of their workday in front of screens, is negatively impacting patient care, in-person interactions, and their professional identities [79]. Therefore, clear communication guidelines and policies fostering minimal disruption during time-boxed discussions compounded by initiatives such as restricting email access, optimizing inbox management, and adhering to an appropriate email etiquette can significantly boost productivity while diminishing stress levels [67]. Moreover, the absence of standardized patient care paths complicates digital tool design, as health care technology often disrupts, rather than streamlines, workflows. In addition, numerous HCPs work in a hybrid manner, using both digital tools and paper-based processes in parallel, leading to significant inconsistencies [80]. In addition, technical and uncommunicated IT maintenance; system reboots, freezes, and crashes; and full blackouts disrupt clinical workflows, as these result in significant downtime and sometimes a return to paper-based processes.

### The Opportunities and Challenges of Shadow IT

In addition, our study revealed that HCPs have many valuable insights in the digital domain, which they implement mainly through shadow IT. Due to limitations in existing digital tools and gaps, HCPs seek and develop their own solutions to fulfill their workflow requirements. Within this context, HCPs increasingly use unauthorized hardware and software to streamline processes, such as document imaging, scanning, and quick reference retrieval, as well as to promote more efficient collaboration, patient communication, and access to medical knowledge. This adaptive approach showcases the agility of HCPs in leveraging available resources; however, the associated security risks should not be disregarded. Other studies have revealed that health care institutions are grappling with the rise of shadow IT to complement shortcomings in hospital-provided IT resources, which can be beneficial, but also introduces vulnerabilities and potential access points for cyber threats [25]. Generative AI models and social media, along with chat apps, are now vital for information access, while also being used for exchanging sensitive patient data. The exponential increase in chat app use has improved communication but often occurs without HCPs’ awareness of existing privacy and security regulations [81]. Although shadow IT is recognized, HCPs rather discreetly use it, showcasing their dedication to improving efficiency. This innovative yet potentially risky approach underscores the imperative of addressing technological gaps and advancing hospital technology, signaling the need for change. Recognizing where workflow optimizations is reached through unauthorized tools can be of great interest for hospitals as well as HIT developers to inform workflow-aligned innovations for the central IT provision, as well as to identify vulnerabilities and access points for cyberthreats [25].

### Limitations

This interview study has not only notable strengths but also limitations. It addresses gaps in the existing evidence by examining HCPs’ experiences with digital tools in hospital settings. Extensive interviews with a diverse set of HCPs were conducted, offering a comprehensive overview of the digital tool landscape, rather than focusing solely on a specific type of tool or a specific workflow context. The rigorous approach ensured credibility through participant engagement, researcher reflexivity, and transparent data collection and analysis. Moreover, dependability was maintained by applying a literature-derived framework for data comprehension. However, convenience sampling might have introduced a self-selection bias, potentially impacting participants’ attitudes and expectations. In addition, as the data are solely derived from interviews, these might lack structured observations of HCPs’ interactions with digital tools. Besides this, the focus on hospital settings may limit generalizability to the primary care context, and perspectives of other stakeholders such as patients were only indirectly conveyed through HCPs. Finally, the study’s location in Switzerland may constrain its applicability to other health care systems. Subsequent research could explore more discipline- or tool-specific insights as well as other contexts and health care environments.

### Conclusions

Our study was designed to assess the experience of HCPs working with digital tools in hospitals. Despite the acknowledged benefits and possibilities offered by digital technologies in hospitals, these tools place a daily burden on HCPs that must be addressed. Within the ongoing digital transformation of almost every health care institution, significant paradoxes and conflicting perspectives regarding the optimal strategy for HIT development and implementation exist. Stakeholders must rethink the construction of digital tools and hospital IT systems and challenge the prevailing status quo. Health care tools and systems should be designed with flexibility and adaptability at their core to meet the diverse requirements of users in various contexts and should adopt a lightweight approach, enabling them to adapt to advancements and allowing local tailoring. To achieve this, a sufficient implementation strategy is crucial, and robust evaluation methods for these technologies are necessary, emphasizing a thorough examination of users’ needs and the contexts in which they operate. Effective and tailored training is another critical aspect of ensuring that HCPs are equipped with the necessary skills to harness the potential of digital tools and data together with addressing the issue of information overload through improved communication guidelines.

In this study, paradoxes emerged from HCPs’ perspectives, often highlighting opposing forces within reported experiences. Nevertheless, clinician experiences are frequently neglected in projects that aim to address HIT issues. Historically, emphasis has been placed on IT and commercial teams, relegating clinician insights more toward the periphery. However, the paradoxes identified from clinicians’ viewpoints can provide invaluable insights that suggest new strategies to navigate paradoxes and address enduring challenges.

## Appendix

Multimedia Appendix 1 COREQ (Consolidated Criteria for Reporting Qualitative Research): 32-item checklist.

Multimedia Appendix 2 Semistructured interview guide.

Multimedia Appendix 3 Additional tables with quotation counts and supportive quotes.

## Abbreviations

AI: artificial intelligence
COREQ: Consolidated Criteria for Reporting Qualitative Research
EHR: electronic health record
HCP: health care professional
HIT: health information technology

## Data Availability

Full interview transcripts and audio files are not publicly available due to data protection reasons; however, metadata, including the codebook and extracts that support the findings, are available on a Zenodo repository [82].
