# Supplementary material for: The Paradoxes of Digital Tools in Hospitals: Qualitative Interview Study
Source: J Med Internet Res. 2024 Jul 15;26:e56095. doi: 10.2196/56095 (PMC11287097; doi:10.2196/56095)
Supplement: Multimedia Appendix 2 [file jmir_v26i1e56095_app2.docx]

## Semistructured interview guide to assess health care professionals’ experience of using digital tools in the hospital

**ENGLISH VERSION**

| **Interview set-up** | | **Insights & objective** | | |
| --- | --- | --- | --- | --- |
| **Guiding research questions** | What is the experience of healthcare professionals using digital tools in hospitals in Switzerland? | *The point of a qualitative interview is to let the respondent tell their own lived experiences and stories on their own terms.* | | |
| **Methodology** | This qualitative research study is conducted through in-person interviews with healthcare professionals (physicians and nurses) in Switzerland, lasting between 45-60 min, that aims to understand the experience with digital tool/s in hospital settings. HCPs are required to have at least 6 months of experience with the digital tool/s and can report this experience in English or German. The interviews will take place in person at the respective hospital sites.  To understand the HCPs’ experiences with digital tool/s, the questions that are asked are guided by two theoretical frameworks, i.e., the Technology Acceptance Model (TAM) and the Work System Model (WSM) [27,28].  While TAM is used to understand and explain how HCPs perceive and adopt new technologies, the WSM helps to explore how people, processes, and technology interact within a working system to identify the different components of the healthcare work system and how they affect HCPs’ use of digital tool/s [27,28]. This model also helps to uncover potential barriers and facilitators to the adoption of digital tool/s in healthcare. | *Using frameworks to guide the interview questions helps ensure that the data collected is relevant, organized, and can be analyzed in a meaningful way.* | | |
| **Interview guide** | | **Insights & objective** | **Allocated time min)** | **Total (min)** |
| **Personal introduction** | Good morning/ afternoon/ evening, my name is Marie Wosny, I am a Ph.D. student at HSG and UZH, and interested in learning about digital tool/s and their use in hospital settings […].  Good morning/ afternoon/ evening, my name is Janna Hastings, and I am a Professor at HSG and UZH, […].  I will be conducting this interview with you today. Thank you for taking the time to participate in this study.  As the interviewer and moderator, my role is to guide this conversation and ask you questions related to your experience with digital tool/s in hospital settings. I will use this paper-based interview guideline to ensure that we cover all relevant topics.  I will be listening attentively to your responses and may ask follow-up questions as needed to ensure that we fully understand your thoughts and opinions on the topic.  Additionally, I’ll be keeping track of time to ensure that we cover all the questions in an approx. 45-minute time frame.  May I ask if you permit us to record the interview? The recording will not be released to anyone, but we will transcribe it for analysis.  Thank you for your confirmation, I will start the recording now. | *Greet the interviewee and introduce each other* | 10 | 10 |
| **Study introduction** | Before we begin, I would like to explain the purpose of this interview: I will be asking you questions to understand your experience of using digital tool/s in your day-to-day practice.  Your responses will be kept anonymous and treated confidentially, and you have the right to withdraw from this research at any time.  This research will be carried out within ethical guidelines and direct quotes will only be used anonymously to illustrate findings.  I kindly ask that you refrain from sharing any specific patient experiences or clinical data, including adverse events.  We will now start the interview. | *Explain the overall flow and agenda* | 2 | 12 |
| **Background information, eligibility, and screening** | Before we begin discussing your experience with digital tool/s in hospital settings, I would like to ask a few questions to better understand your background and experience as a healthcare professional.  This will help me contextualize your responses and better understand your perspective.  *Sign-up form (already addressed)*  *Name*  *Biological gender*  *Current position*  *Education level (senior vs. assistant)*  *Hospital affiliation (indirect location, size, setting)*  *Specialization/field of expertise*  Screening and background questions  Could you please take a moment to introduce yourself and tell us a little bit about your professional background?  Would you be so kind and tell us your age and employment status (full-time, part-time, etc.) | *Identification of eligible study participants was conducted through the interview sign-up form. Further screening questions provide a basic demographic profile of HCPs.* | 5 | 17 |
| **Topic** | **Guiding question (general)** | **Possible follow-up question (more specified)** |  |  |
| **WSM: Technology & information:** | How often do you use digital tools in your practice, and for what purposes?  When did you start using the/se tool/s for the first time? | Which tool do you use the most?  Which tool do you like the most?  Otherwise: pick one you would like to report on  Are there any digital tool/s or software that you are particularly skilled with?  Have you received adequate training and support for using the/those tool/s? | 5 | 22 |
| **WSM: Process** | Can you describe the clinical workflows or tasks that involve digital tool/s in your practice? | Have you noticed any changes in the efficiency or effectiveness of those workflows since implementing digital tool/s?  Have there been any challenges or obstacles to using digital tool/s in your clinical workflows, and how have you addressed them? | 3 | 25 |
| **WSM: People** | Have digital tools impacted your interactions with colleagues? If so, how?  Have digital tools impacted your interactions with patients? If so, how? | Have you noticed any changes in your roles or responsibilities because of using digital tools? | 5 | 30 |
| **Technology Acceptance Model (TAM)**: *Perceived usefulness, perceived ease of use, and subjective norms* | How useful do you find the digital tool/s you use in your practice?  Do you find the digital tool/s you use to be easy to learn and use? | Have you received support or encouragement from colleagues or peers to use digital tool/s in your practice?  To what extent do you perceive the use of digital tool/s as voluntary or mandatory in your daily work? | 3 | 33 |
|  | Was there a tool that has been introduced to you that you should have been using but did not, and if so, why? |  | 2 | 35 |
| *Moving on* | Thank you for the interesting discussion so far.  To dive deeper into your personal emotional experience with the/se tool/s, I would like to shift our focus to your personal feelings and thoughts. | *N.A.* | 1 | 36 |
| **Focus on positive feelings and thoughts** | I am curious to know how you feel when you use digital tool/s in your practice.  Have you experienced any positive emotions or feelings when using digital tool/s, and if so, can you describe them?  Can you please describe one example in detail? | Please tell me more.  What do you mean?  If they struggle to identify any: “Positive emotions people sometimes feel are confidence, responsible satisfaction, gratefulness, or joy. Do any of these describe how you felt?” | 5 | 41 |
| **Focus on negative feelings and thoughts** | Have you experienced any negative emotions or feelings when using digital tool/s, and if so, can you describe them?  Can you please describe one negative example in detail? | Please tell me more.  What do you mean?  If they struggle to identify any: “Negative emotions people sometimes feel are frustration, feeling overwhelmed or discouraged, fear, confusion, or sad. Do any of these describe how you felt?” | 5 | 46 |
| **Further details** | Do you feel confident and comfortable using digital tool/s in your practice, or do you have any concerns or reservations?  Have you ever felt frustrated or overwhelmed by the digital tool/s you use, and if so, can you describe what led to those feelings?  In general, do you trust the tools, and if so, why, or why not?  Do these tools affect how you feel about your work or yourself as a [XXX] (their job role, e.g., physician/nurse) | How did it affect your work experience? | 5 | 51 |
| **Closing question:** *Leave the respondent feeling empowered* | Based on your experience, what advice would you give to other healthcare professionals who are just starting to use the/digital tool/s in their practice? | In your opinion, what are some of the best practices for using digital tool/s effectively in a healthcare setting? | 3 | 53 |
| **Open questions:** *Allow the interviewee to address any questions or gaps* | Thank you so much for a great discussion.  Before we wrap up, please let us know if there is any important point missing that we have not asked you?  Is there anything else you would like to share with us? | *N.A.* | 2 | 55 |
| **Wrap-up:** Thank the interviewee for their time and let them know what the next steps in the process will be | That concludes our interview.  Thank you so much for taking the time to share your thoughts and experiences with me. Your insights will be incredibly valuable in helping us understand how HCPs are using digital tool/s in the hospital  We will be sending you a transcription of our conversation in the next few days.  This will give you an opportunity to review our discussion and make any necessary changes or corrections.  Do you have any questions or concerns about the interview or the transcription process? | *N.A.* | 2 | 57 |
| **Snowballing sampling technique** | One last question from our side: Do you know of any colleagues who have experience using digital tool/s in their work that you think would be willing to participate in this study?  We are looking to speak with a diverse range of individuals and any referrals would be greatly appreciated.  Thank you.  I will now stop the recording | *N.A.* | 2 | 59 |
| ***Stop recording*** | | | | |
| **Goodbye** | Thank you again for your time and insights.  It was a pleasure speaking with you today.  Take care and all the best. | *N.A.* | 1 min | |

**GERMAN VERSION**

| **Interview Rahmenbedingungen** | | **Begründung und Zielsetzung** | | |
| --- | --- | --- | --- | --- |
| **Leitende Forschungsfrage** | Welche Erfahrungen machen Ärzte:innen und Pflegefachpersonal bei der Nutzung digitaler Tools in der Klinik in der Schweiz? | *Der Zweck eines qualitativen Interviews ist es, den Befragten ihre eigenen gemachten Erfahrungen spezifisch auf ihre eigene persönliche Weise erzählen zu lassen.* | | |
| **Methode** | Im Rahmen dieser qualitativen Forschungsstudie werden Ärzte:innen und Pflegefachpersonal in Schweizer Spitälern zu ihren Erfahrungen mit digitalen Tools in der Klinik befragt. Hierbei sind persönliche 45-60-minütige Interviews geplant, um ein tiefes Verständnis der Thematik zu erlangen. Teilnehmende müssen über mindestens 6 Monate Erfahrung mit den digitalen Tools verfügen und ihre Erfahrungen auf Englisch oder Deutsch schildern können. Die Interviews werden persönlich in den jeweiligen Spitälern stattfinden  Um ein Verständnis für die Erfahrungen von Medizinner:innen und Pflegefachkräften mit digitalen Tools zu erlangen, werden die Interviewfragen von zwei theoretischen Frameworks, dem Technology Acceptance Model (TAM) und dem Work System Model (WSM), abgeleitet [27,28].  Das TAM wird genutzt, um zu verstehen, wie Ärzte:innen und Pflegefachpersonal neue Technologien wahrnehmen und übernehmen [27]. Das WSM unterstützt dabei, zu erforschen, wie Menschen, Prozesse und Technologie innerhalb eines Arbeitssystems interagieren, um die verschiedenen Komponenten der Klinik zu identifizieren und zu verstehen, wie diese die Nutzung, Einführung und Annahme von digitalen Tools beeinflussen [28]. | *Durch die Verwendung von “Frameworks” zur Orientierung der Interviewfragen kann sichergestellt werden, dass die gesammelten Daten relevant, organisiert und auf sinnvolle Weise analysiert werden können.* | | |
| **Interview Leitfaden** | | **Begründung und Zielsetzung** | **Zeitein- teilung (min)** | **Total (min)** |
| **Persönliche Vorstellung** | Guten Morgen/Tag/Abend, mein Name ist Marie Wosny, ich bin Doktorandin an der HSG und UZH und interessiere mich dafür, wie digitale Tools im klinischen Alltag genutzt werden […].  Guten Morgen/Tag/Abend, mein Name ist Janna Hastings, ich bin Professorin an der HSG und UZH […].  Ich werde heute dieses Interview mit Ihnen führen. Vielen Dank, dass Sie sich die Zeit genommen haben, an dieser Studie teilzunehmen.  Als Interviewerin und Moderatorin ist es meine Rolle, dieses Gespräch zu leiten und Fragen zu stellen, die sich auf Ihre Erfahrungen mit digitalen Tools in der Klinik beziehen. Dafür werde ich diesen Interview Leitfaden nutzen, um sicherzustellen, dass wir alle relevanten Themen abdecken.  Auf Ihre Antworten werde ich gegebenenfalls weitere Fragen stellen, um sicherzugehen, dass wir Ihre Gedanken und Meinungen zum Thema vollständig verstehen.  Zusätzlich werde ich darauf achten, dass wir innerhalb des Zeitrahmens von ca. 45 Minuten alle Fragen abdecken können.  Wäre es für Sie in Ordnung dieses Interview nun aufzuzeichnen? Die Aufnahme wird vertraulich behandelt und niemandem zur Verfügung gestellt, sondern nur von uns intern transkribiert und für die weitere Analyse genutzt.  Vielen Dank für Ihre Erlaubnis und Bestätigung, ich werde jetzt mit der Aufnahme beginnen. | *Begrüssung des Gesprächspartners und gegenseitige Vorstellung* | 10 | 10 |
| **Einführung in die Forschungsstudie** | Bevor wir mit dem Interview beginnen, möchte ich kurz den Ablauf dieses Interviews erklären: Ich werde Ihnen Fragen stellen, um Ihre Erfahrung bei der Verwendung digitaler Tools in Ihrer täglichen Praxis zu verstehen.  Ihre Antworten werden anonymisiert und vertraulich behandelt, und Sie haben das Recht, sich jederzeit aus diesem Interview auszutreten oder Antworten zurückzuziehen.  Unsere Forschungsstudie wird gemäss ethischer Richtlinien durchgeführt, und direkte Zitate werden nur anonym verwendet, um Ergebnisse zu veranschaulichen.  Ich bitte Sie während des Interviews keine spezifischen Patienten-erfahrungen oder klinischen Daten, einschliesslich Nebenwirkungen, zu teilen.  Wir werden nun mit dem offiziellen Interview beginnen. | *Erklärung des allgemeinen Ablaufs und der Richtlinien* | 2 | 12 |
| **Hintergrund-informationen, Teilnahme-kriterien und Screening** | Bevor wir über Ihre Erfahrungen mit digitalen Tools im klinischen Umfeld sprechen, möchte ich Ihnen ein paar Fragen stellen, um Ihren Hintergrund und Ihre Erfahrung als Ärztin/Arzt/Pflegefachkraft besser zu verstehen.  Dies wird uns dabei helfen, Ihre Antworten in den richtigen Kontext zu setzen und Ihre Perspektive besser zu verstehen.  *Anmeldeformular (bereits aufgenommen)*  *Name*  *Biologisches Geschlecht*  *Aktuelle Jobposition*  *Ausbildungsabschluss (Leitung vs. Assistent)*  *Spitalzugehörigkeit (indirekte Standortangabe, Organisationsgrösse und Ausgangslage)*  *Spezialisierung/Fachgebiet*  Screening and Hintergrundinformation  Könnten Sie sich bitte einen Moment Zeit nehmen, um sich vorzustellen und uns ein wenig über Ihren beruflichen Hintergrund zu erzählen?  Wären Sie so freundlich und würden uns Ihr Alter und Ihren Beschäftigungsstatus (Vollzeit, Teilzeit, usw.) mitteilen? | *Die Identifizierung der geeigneten Studienteilnehmer wurde bereits über Grundfragen im Anmeldeformular für das Interview durchgeführt. Weitere Screening-Fragen liefern Informationen für das grundlegende demografische Profil von den Ärzte:innen und Pflege-fachkräften* | 5 | 17 |
| Thema | Leitfrage (allgemein) | Mögliche Follow-up Frage (spezifisch) |  |  |
| **Warm-up Frage zu WSM-Technologie & Informationen*:*** *Offene und einfach zu beantworte Frage, um eine angenehme Atmosphäre zu schaffen und den Übergang zum Hauptteil des Interviews zu erleichtern, sowie für die Vertrauens-bildung zwischen dem Interviewer und dem Interview-partner* | Wie oft nutzen Sie digitale Tools in Ihrer Praxis und zu welchen Zwecken?  Wann haben Sie die/dieses Tool/s zum ersten Mal verwendet? | Welches Tool verwenden Sie am häufigsten?  Welches Tool gefällt Ihnen am besten?  Ansonsten: Wählen Sie ein Tool aus, über das Sie berichten möchten.  Gibt es digitale Tool/s oder Software, mit denen Sie besonders vertraut sind?  Haben Sie eine angemessene Schulung/Training und Unterstützung für die Verwendung des/dieser Tool/s erhalten? | 5 | 22 |
| **WSM: Prozess** | Können Sie bitte die klinischen Workflows oder Aufgaben beschreiben, bei denen digitale Tools in Ihrer Praxis verwendet werden? | Haben Sie Veränderungen in der Effizienz oder Effektivität dieser Workflows festgestellt, seitdem digitale Tools implementiert wurden?  Gab es Herausforderungen oder Hindernisse bei der Verwendung von digitalen Tools in Ihren klinischen Workflows und wie gehen Sie mit diesen um? | 3 | 25 |
| **WSM: Menschen** | Haben sich digitale Tools auf Ihre Interaktionen mit Kollegen ausgewirkt? Wenn ja, wie?  Haben sich digitale Tools auf Ihre Interaktionen mit Patienten ausgewirkt? Wenn ja, wie? | Haben Sie Veränderungen in Ihrer professionellen Rolle oder in Verantwortlichkeiten bemerkt, die auf die Verwendung der digitalen Tools zurückzuführen sind? | 5 | 30 |
| **TAM:** *Wahrgenommene Nützlichkeit, wahrgenommene Benutzerfreundlichkeit und subjektive Normen* | Als wie nützlich bewerten Sie die digitalen Tools, die Sie in Ihrer täglichen Praxis verwenden?  Finden Sie die digitalen Tools, die Sie benutzen, einfach zu erlernen und zu verwenden? | Bekommen Sie Unterstützung oder Fürsprache von Kollegen oder, digitale Tools in Ihrer Praxis zu verwenden?  Inwieweit empfinden Sie die Verwendung von digitalen Tools in Ihrer täglichen Arbeit als freiwillig oder verpflichtend? | 3 | 33 |
| **Ausschluss von Tools** | Gab es ein Tool, das bei Ihnen vorgestellt und eingeführt wurde, dass Sie eigentlich hätten verwenden sollen, aber dieses nicht verwendet haben, und wenn ja, warum? |  | 2 | 35 |
| ***Fortführung zum nächsten Teil*** | Vielen Dank für die bisher sehr interessante Diskussion.  Um tiefer in Ihre Erfahrungen mit dem/den Tool/s einzutauchen, möchte ich unseren Fokus auf Ihre persönlichen Gefühle und Gedanken beim Nutzen der Tools lenken. | *N.A.* | 1 | 36 |
| **Fokus auf positive Gefühle und Gedanken** | Ich würde gerne erfahren, wie Sie sich fühlen, wenn Sie digitale Tool/s in Ihrer Arbeit verwenden.  Haben Sie beim Einsatz digitaler Tool/s irgendwelche positiven Emotionen oder Gefühle erlebt und wenn ja, können Sie diese beschreiben?  Können Sie bitte ein korrektes positives Beispiel detailliert beschreiben? | Bitte erzählen Sie mehr.  Wie meinen Sie das?  Falls sie Schwierigkeiten haben, eine Antwort zu finden: Positive Emotionen, die Menschen manchmal empfinden, sind Selbstvertrauen, Verantwortlichkeit, Zufriedenheit, Dankbarkeit oder Freude. Beschreiben einige dieser Emotionen vielleicht, wie Sie sich gefühlt haben?” | 5 | 41 |
| **Fokus auf negative Gefühle und Gedanken** | Haben Sie beim Einsatz digitaler Tool/s irgendwelche negative Emotionen oder Gefühle erlebt und wenn ja, können Sie diese beschreiben?  Können Sie bitte ein konkretes negatives Beispiel detailliert beschreiben? | Bitte erzählen Sie mehr.  Wie meinen Sie das?  Falls sie Schwierigkeiten haben, eine Antwort zu finden: „Negative Emotionen, die Menschen manchmal empfinden, sind Frustration, ein Gefühl von Überwältigung oder Entmutigung, Angst, Verwirrung oder Traurigkeit. Beschreiben einige dieser Emotionen vielleicht, wie Sie sich gefühlt haben?” | 5 | 46 |
| **Weitere Details** | Fühlen Sie sich sicher und wohl beim Gebrauch digitaler Tool/s in Ihrer Arbeit oder haben Sie Bedenken oder Vorbehalte?  Haben Sie sich jemals durch die digitalen Tool/s, die Sie verwenden, frustriert oder überfordert gefühlt und wenn ja, können Sie beschreiben, was zu diesen Gefühlen geführt hat?  Im Allgemeinen, vertrauen Sie diesen Tools und wenn ja, warum oder warum nicht?  Beeinflussen diese Tools, wie Sie sich über Ihre Arbeit oder über sich selbst als [Berufsbezeichnung] denken? | Wie hat sich dies auf Ihre Arbeitsumgebung ausgewirkt? | 5 | 51 |
| **Schlussfrage:** *Die Teilnehmer sollen sich gestärkt und selbstbewusst, positiv fühlen* | Welche Ratschläge würden Sie basierend auf Ihren Erfahrungen an andere Kollegen:innen geben, die gerade erst damit beginnen, diese/s digitale Tool/s zu verwenden? | Was sind Ihrer Meinung nach die “Best Practices” für die Nutzung von digitalen Tools in der Klinik? | 2 | 53 |
| **Offene Fragen:** *Möglichkeit für die Teilnehmenden, um Lücken oder Fragen zu adressieren* | Vielen Dank für das grossartige Gespräch.  Bevor wir das Interview beenden, würden wir Sie gerne fragen, ob wir in Ihren Augen wichtige Punkte ausgelassen, die wir Sie nicht gefragt haben?  Gibt es sonst noch etwas gibt, was sie uns mitteilen möchten? | *N.A.* | 2 | 55 |
| **Wrap-up:** *Bedanken bei den Teilnehmenden und Informationen die nächsten Schritte im Prozess* | Damit können wir unser Interview beenden.  Vielen Dank, dass Sie sich die Zeit genommen haben, Ihre Gedanken und Erfahrungen mit uns zu teilen. Ihre Erkenntnisse sind sehr wertvoll und helfen uns zu verstehen, wie digitale Tools in der Klinik eingesetzt werden.  Wir werden Ihnen in den nächsten Tagen eine Transkription unseres Interviews zur Einsicht zusenden.  Dies gibt Ihnen die Möglichkeit, unser Gespräch zu überprüfen und etwaige Änderungen oder Korrekturen vorzunehmen.  Haben Sie noch Fragen oder Bedenken hinsichtlich des Interviews oder des Transkriptionsprozesses? | *N.A.* | 2 | 57 |
| **Snowballing Sampling Technik** | Eine letzte Frage von unserer Seite: Kennen Sie Kollegen, die ebenfalls Erfahrung mit der Verwendung von digitalen Tools haben und eventuell bereit wären, an dieser Studie teilzunehmen?  Wir möchten mit einer vielfältigen Gruppe von Personen sprechen und jede Empfehlung wäre sehr willkommen.  Wie können wir diese Kollegen am besten kontaktieren?  Vielen Dank.  Ich werde die Aufnahme jetzt stoppen. | *N.A.* | 2 | 59 |
| ***Stoppen der Aufnahme*** | | | | |
| Verabschiedung | Nochmals vielen Dank für Ihre Zeit und Teilen Ihrer Erfahrungen.  Es war eine Freude heute mit Ihnen zu sprechen.  Alles Gute und auf Wiedersehen. | *N.A.* | 1 min | |
