# Supplementary material for: The Paradoxes of Digital Tools in Hospitals: Qualitative Interview Study
Source: J Med Internet Res. 2024 Jul 15;26:e56095. doi: 10.2196/56095 (PMC11287097; doi:10.2196/56095)
Supplement: Multimedia Appendix 3 [file jmir_v26i1e56095_app3.docx]

**Additional tables with quotation counts and supportive quotes.**

**Table S1.** Commonly reported digital tool strengths, deficits, requirements, and unauthorized resources (number of study participants).

| **Tool strengths** | **Tool deficits** | **Tool requirement** | **Unauthorized IT resources** |
| --- | --- | --- | --- |
|  |  |  |  |
| Remote access (15) | Interoperability issues (21) | Comprehensive, customizable, and integrative solution (18) | Use of personal hard- and software (12) |
| System interoperability (14) | Technical malfunctions (21) | Automation functions (14) | Use of chat apps (8) |
| Automation function (11) | Counterintuitive tool design (18) | Result-oriented design (9) | Use of large language models (3) |
| Provision of comprehensive overview (8) | User unfriendly tool design (17) | Adoption to situation and discipline (6) | Use of social media platforms (2) |
| Intuitive tool design (7) | Incompatible systems (17) | Balances level of information (4) | Use of video platforms (2) |
| Manual corrections possible (7) | Various specific and individual tools (17) | Engaging visualization (4) |  |
| Automated data transfer (6) | Cumbersome tool visualization (13) | Fit for purpose (4) |  |
| Decision support function (5) | Cumbersome tool design (13) | Structured and logical tool design (4) |  |

**Table S2.** Supporting quotes on reported digital tool attributes, issues, and requirements.

| **Topic** | **Reported quote** (role of study participant) |
| --- | --- |
|  |  |
| Digital tool demands based on medical discipline | *“Medication prescriptions and patient records are digitized, this is actually exceptional for the intensive care unit as compared to the internal medicine department.” (Resident physician)* |
|  | *“In the Intensive Care Unit everything is completely electronic, but there is no interface with our hospital system.” (Senior nurse)* |
|  | *“Here in the Emergency Department, you must always be able to perform tasks with just a few clicks since you can only access a computer for a short amount of time. It’s quite different as compared to another wards or departments.” (Senior nurse)* |
| Access to data and information | *“Access to information is now quicker and more efficient. If you can find a computer somewhere, you can log in and retrieve the necessary information. You don’t have to search all over the place anymore. Particularly in radiology, there has been a lot of progress. In the past, we used to race through the building every day with paper folders and analog X-rays. I believe the speed of accessing information has improved for the benefit of patients.” (Senior physician)* |
|  | *“It is highly appreciated, especially in interprofessional collaboration. We have recently introduced a nursing dossier, where we compile interprofessional documents within the current clinical information system. It includes nursing histories that also encompass social background. The objective is to provide doctors and social services with access to all relevant data, which is a significant advantage.” (Senior nurse)* |
|  | *“In the clinical information system, there are various options where specific findings can be stored. There are three different locations for this purpose. You might assume it’s typically in one place, but then it suddenly turns up in another. So practically, if you can’t find something, you need to search in different locations because it could potentially be stored anywhere.” (Resident physician)* |
| Digital tool strengths | *“I’ll begin at home, where I have remote access. There, I can use the same interface as the one available at the hospital, and I find that very beneficial.” (Senior physician)* |
|  | *“One of the aspects I truly appreciate about the clinical information system is that everything functions within a single program. You do not need to open any external applications.” (Resident physician)* |
|  | *“What I found particularly advantageous was the seamless transfer of data from the surveillance monitor. It’s reassuring that you don’t have to painstakingly click through; it all happens automatically. That’s a huge relief.” (Staff nurse)* |
| Digital tool deficits | *“I find it quite cumbersome because there are three different programs that don’t communicate with each other.” (Resident physician)* |
|  | *“Moreover, the need to switch between these programs is inconvenient. They don’t integrate smoothly, and we’re certain that the key connection points between these systems are not 100% compatible. This often leads to issues.” (Staff nurse)* |
|  | *“When a problem arises the initial answer is often, “We make a tool for that”. Subsequently, someone, usually at a significant cost, begins developing a tool. However, it’s often rare to see truly effective, streamlined, and good results.” (Senior physician)* |
|  | *“The clinical information system is a quite unstable program. It crashes regularly, even during the visits, and that’s really annoying.” (Resident physician)* |
|  | *“The system has limitations in terms of space. You’re confined to a single page for recording diagnoses, and once that page is full, there’s no additional space. Consequently, you have to reduce the font size to the point where it becomes unreadable.” (Resident physician)* |
| Digital tool requirements | *“For me, it would be that I could ideally access or integrate everything from a future clinical information system.” (Senior nurse)* |
|  | *“And automate as many administrative processes as possible. That helps when you no longer have to do them.” (Senior physician)* |
|  | *“It is also important to simply adapt processes or systems appropriately so that, at the end of the day, it somehow benefits the employees, the patients, or ideally both.” (Senior nurse)* |
| Decision-making on digital tool implementation | *“We initially tried [to improve the situation], and we couldn’t resist, because it was a decision imposed from management. However, later on, we had to report that it wasn’t effective. Another IT-related aspect of this institution is the telephone service, which is, in fact, not functional. Nevertheless, I have to use it, there’s no alternative or choice.” (Senior nurse)* |
|  | *“Most of the time, these are tools that one must utilize. You cannot say “I won’t engage in performance tracking because I dislike the tool or because it doesn’t function a certain way”. The reality doesn’t allow for such an approach.” (Senior physician)* |
|  | *“There are certain tools that I don’t use, but with the ones I’m required to use, I must adhere to them. There isn’t any instance where I have the liberty to decide not to use them.” (Resident physician)* |
| Trust in digital tool | *“What I find complicated are the things with a ‘black box’ where you can’t see how the analysis is done or understand how it works. In those cases, you have to rely solely on the measurement results.” (Senior physician)* |
|  | *“I would say it depends on the user whether they do things correctly and fill out forms accurately.” (Resident physician)* |
|  | *“I trust that the IT department ensures security and maintains good backups. So, I do have trust in the tools, even though it’s not 100% secure. I’m aware of that, but I still have confidence in them.” (Senior physician)* |

**Table S3.** Commonly reported workflow improvements and deterioration by digital tools (number of study participants).

| **Workflow improvement** | **Influencing factors for workflow improvement** | **Workflow deterioration** | **Influencing factors (or workflow deterioration** |
| --- | --- | --- | --- |
|  |  |  |  |
| Gain in efficiency (37) | Good workflow integration (22) | Time consumption (34) | Hybrid work: paper and electronic (23) |
| Time saving (34) | Helpful and supporting tool (17) | Inefficiencies (22) | Cumbersome processes (22) |
| Increased productivity (8) | Full care path and workflow correctly mapped (10) | More effort/cost than benefit (11) | Long waiting times for loading or booting (19) |
| Better time resource management (6) | Remote access (5) | More distraction and less focus (9) | Suboptimal workflow integration (15) |
| Decreased workload (6) | Sufficient system functionality (5) | No measurable outcome (9) | Processes incorrectly mapped (10) |
| Mental support and assurance (4) | Less phone calls (4) | Overflow of information (8) | E-mail overflow (9) |
| Learning effect (3) | Template text blocks (4) | Work hindered (7) | Redundant or repetitive processes (9) |

**Table S4.** Supporting quotes on reported workflow improvements and deterioration by digital tools.

| **Topic** | **Reported quote** (role of study participant) |
| --- | --- |
|  |  |
| Impact of digital tools on collaboration | *“Now, we can all access and work with the data as a team, which has significantly changed how we work as physicians. We have this great ability to access and modify data, among other things.” (Resident physician)* |
|  | *“I receive a large number of e-mails every day, but most of them aren’t relevant for my work. So, I am getting around 100 emails daily and I have to figure out which ones are important, which ones need attention, and which can be deleted. This process takes up a lot of time and doesn’t provide much benefit. Additionally, the threshold for sending emails is just too low.” (Senior physician)* |
|  | *“Patients might visit their primary care physician or a nearby specialist before returning to us. Unfortunately, the data exchange in such cases is remarkably inefficient. We are presently scanning and uploading external PDFs, which is simply inadequate for today’s standards. It must operate differently in this modern era, as this is undoubtedly a significant drawback.” (Senior nurse)* |
| Impact of digital tools on workflow deterioration | *“Working with this daily program can be quite frustrating due to its inherent inefficiency.” (Resident physician)* |
|  | *“And then the phone rings, pulling you out of your workflow once again. You’re compelled to perform specific tasks on your phone, and only after completing those tasks can you return to your initial workflow. I believe this is a significant source of errors.” (Staff nurse)* |
|  | *“However, I strongly believe that one’s efficiency increases when interruptions are minimized. [...] Especially during team meetings, I’ve noticed that about half of the team, while someone is speaking, tends to look at their phones or engage in other activities because they have received a message. This affects our interpersonal interactions, which are already rare. When we do have the chance to meet, it’s crucial that we engage in meaningful conversations and exchange ideas without distractions. Unfortunately, I’ve observed that questions are often repeated, and important points get lost because some colleagues are distracted by notifications, messages, or e-mails on their phones, diverting their attention from the discussion. Consequently, our morning meetings can easily become extended due to these digital distractions.” (Senior physician)* |
| Impact of digital tools on workflow improvement | *“So, the patient curve generated electronically is identical to the one produced manually. That’s excellent.” (Resident physician)* |
|  | *"It’s also a significant advantage that you can securely access information from different locations. For instance, in terms of planning, I can create the daily schedule from my office, while someone at the station office can simultaneously update that a patient is being discharged. I can instantly see these updates even when I’m not physically on-site." (Senior nurse)* |
|  | *“We have transitioned into a nearly paperless clinic. Our entire workflow has been digitized with the assistance of Care Paths defined within our IT infrastructure. Every aspect of our operations has been successfully integrated.” (Resident physician)* |
| Burden of administrative tasks and bureaucracy | *“About 80% of our time is spent on documentation and only 20% for direct patient care. A significant amount of time goes into documentation or organizing tasks.” (Resident physician)* |
|  | *“Currently, spending 30% of our time with patients is considered good, while 70%, or more, is dedicated to phone calls, recording examinations, and writing letters. It’s far from ideal.” (Senior physician)* |
|  | *“I estimate that nearly half of my working hours are spent at the computer, while the remaining 50% are dedicated to patient care.” (Staff nurse)* |
| Conflict between standardization and individualization in medicine | *“You can already observe this dilemma in our healthcare system. Somewhere, there has to be a compromise. When everyone has their own needs and demands, and you attempt to accommodate them all in a single tool, it becomes challenging, and you struggle to achieve good outcomes.” (Senior nurse)* |
|  | *“I lean somewhat towards standardization, but fundamentally, I believe that many aspects of medicine do not adhere to a one-size-fits-all approach.” (Senior physician)* |
|  | *“Data quality is important. When you digitize and automate standardized processes, the data tend to exhibit greater consistency in terms of quality, usually resulting in improvements, and, most importantly, there are no longer individual variations.” (Senior physician)* |

**Table S5.** Commonly reported experiences and outcomes due to the use of digital tools (number of study participants).

| **Negative experience** | **Positive experience** | **Improved patient care** | **Suboptimal patient care** | **Change in professional image** |
| --- | --- | --- | --- | --- |
|  |  |  |  |  |
| Frustrated (46) | Satisfied (14) | Core emphasis and focus on patient care/wellbeing (16) | Patient safety risk (16) | Change (28) |
| Bothered and annoyed (21) | Excited (8) | Patient-centered interactions (15) | Reduced patient interaction time due to documentation (15) | No change (24) |
| Angry (12) | Grateful (7) | Increase in patient safety (14) | Healthcare provider image shift to screen-centric care (13) |  |
| Dissatisfied (12) | Relieved (7) | Better informed patient (12) | Reduced quality of patient care (8) |  |
| Stressed (9) | Confident (5) | Increased quality of patient care (11) | No effects on patient (6) |  |
| Helpless (8) | Hope (5) | Better informed HCP (10) | Patient concerns (3) |  |
| Tedious (8) | Reassured (5) | Patient has access to data (9) | Layperson partial understanding and misinformation (2) |  |

**Table 6.** Supporting quotes on experiences and outcomes due to the use of digital tools.

| **Topic** | **Reported quote** (role of study participant) |
| --- | --- |
|  |  |
| Negative experiences of digital tools usage | *“You get completely blocked. I couldn’t access the clinical information system and I couldn’t work. It’s extremely frustrating.” (Staff nurse)* |
|  | *“And then we have to cut back on patients. Or the resident physicians end up working overtime. When they have to deal with those interface management issues, they can’t be with the patients or leave early. That’s frustrating.” (Senior physician)* |
|  | *“Undoubtedly, this places a heavy psychological burden on us because we’re aware that when the system isn’t functioning, we can’t take care of the patients who are in unstable conditions as they arrive. We must wait until the system is functioning again.” (Staff nurse)* |
| Positive experiences of digital tool usage | *“It’s quite satisfying to swiftly access the data that’s relevant to you, enabling you to perform assessments and make necessary adjustments to therapies or conduct consultations.” (Resident physician)* |
|  | *“I was able to schedule the session on my laptop, and it was incredibly satisfying. While it didn’t reduce stress, it certainly provided a sense of relief.” (Senior nurse)* |
|  | *“The emotions involved were a mix of relief, in terms of time-saving, especially at midnight. Additionally, there was a sense of pride because this system is our creation, stemming from our team’s effort.” (Senior physician)* |
| Positive change of HCPs’ professional image caused by digital tools | *“Yes, you definitely feel more connected to the current trends when you compare yourself with colleagues who are, to put it in an exaggerated way, on the verge of retirement and still rely on their outdated knowledge from a decade ago.” (Senior nurse)* |
|  | *“I think it elevates our professionalism and requires us to embrace new developments in our field.” (Senior nurse)* |
| Negative change of HCPs’ professional image caused by digital tools | *“Currently, I feel a relatively small connection to the role of being a physician. It may sound sad, but it’s a significant issue that I am dealing with at the moment.” (Resident physician)* |
|  | *“Yes, the operation of various machines, the documentation – it all involves a high level of digital tools, and I believe this has an effect on one’s experience. […] I believe this does have a negative impact on me as an individual." (Staff nurse)* |
|  | *“Indeed, I think I was somewhat naive when I entered the medical profession, thinking that I wanted to work closely with people. I became somewhat disheartened when I realized how much of my work is spent on computer tasks.” (Resident physician)* |
| No change of HCPs’ professional image caused by digital tools | *“I grew up with it. Since I started my career, there haven’t been any significant shifts.” (Senior physician)* |
|  | *“I don’t believe so. Nowadays, every modern profession relies on digital tools.” (Senior physician)* |
| Dependencies on digital tools and their provider | *“We are entirely dependent on these tools, 24 hours a day, seven days a week.” (Senior physician)* |
|  | *“If my computer doesn’t work, I could theoretically just go back home.” (Senior nurse)* |
|  | *“We had had a total system failure and that has been horrible. [...] I’m completely reliant on these programs for my daily tasks. In fact, every action I perform with a patient involves computer interaction and I can’t perform any task without it.” (Staff nurse)* |
| Shifts of role expectations and skill needs due to digital tools | *“It always requires a user who is proficient and understands the system’s capabilities and limitations. If it provides certain information, I must also be capable of recognizing when it doesn’t align with what I observe and be able to interpret it.” (Resident physician)* |
|  | *“It’s quite challenging. We have incredibly skilled individuals, with not only software development skills but also programming, medical statistics, and surgery. This combined expertise is invaluable, enabling us to achieve remarkable outcomes.” (Senior physician)* |
| Reduced quality of patient care due to digital tools | *“Yes, there have been situations where I couldn’t find crucial information in the clinical information system. This lack of essential data led to incorrect treatment decisions for a patient.” (Senior physician)* |
|  | *“This situation is detrimental to the patient because valuable information is being lost, and that is a serious issue.” (Resident physician)* |
| Improved quality of patient care and empowerment due to digital tools | *“Some may argue that as a doctor, you should be essential to personally assess a patient, rather than electronically initiating diagnostic tests using the smartphone. The emphasis should be on direct patient evaluation.” (Senior physician)* |
|  | *“I believe it has also had a positive impact because we now consistently have all the information readily available. Patients often ask about their lab results and their current medical status. With everything integrated through digital tools and the clinical information system, it’s much easier to access information. This also enables us to provide patients with more comprehensive updates.” (Senior nurse)* |
| Shift towards screen-centered care due to digital tools | *“However, there’s also a risk of becoming overly reliant on these tools and solely focusing on them. For building trust between patients and doctors, as well as fostering common understanding, face-to-face interactions without the use of technical devices are essential.” (Resident physician)* |
|  | *“Often, you find yourself staring more at the screen than at the person you’re talking to. It’s important to maintain a fast-paced workflow for documentation during visits however when I’m engaged in a conversation with someone, it’s difficult to simultaneously type on the computer, and I believe patients pick up on this and feel a sense of distance.” (Senior physician)* |

**Table S7.** Commonly reported factors on digital tool training (number of study participants).

| **Training provider** | **Training experience** | **Timepoint of training** | **Training format** |
| --- | --- | --- | --- |
|  |  |  |  |
| Learning by doing (33) | Getting used to a tool with time (21) | First week general introduction across disciplines (21) | E-learning (11) |
| Experienced colleagues (27) | General intro is useless and too generic (12) | First week introduction within discipline (7) | Organized training session (10) |
| Trial and error (13) | Responsibility of user (10) | At introduction of tool (6) | Mandatory trainings (4) |
| Senior HCP / ward manager (6) | Easy and intuitive (9) | Prior to work, during studies (1) | Video learnings (4) |
| IT representative (4) | Learning with experience and new things even later (5) |  | Handout/factsheet/manual (3) |
| Manufacturer lessons (4) | Difficult to learn (4) |  | Basic training (2) |
| Implementation project teams (2) | Learning new things even later (4) |  | In-depth training for specialist knowledge (2) |

**Table S8.** Supporting quotes on factors on digital tool training.

| **Topic** | **Reported quote** (role of study participant) |
| --- | --- |
|  |  |
| Ineffective training sessions | *“The training session was so basic and fundamental, it had nothing to do with the work I’m doing.” (Senior nurse)* |
|  | *“They tend to explain far too many things that are simply unnecessary, and you tend to forget them immediately. So, I often wonder why I’ve spent three hours on them.” (Staff nurse)* |
|  | *“At the hospital level, there are mandatory e-learning modules that everyone has to complete. Some of these modules have no relevance to my work, but we are all required to complete them.” (Senior physician)* |
| Effective trainings and experimental learning approaches | *“I find it more beneficial to learn from my colleagues who have to perform the same tasks as I do.” (Senior physician)* |
|  | *“It’s genuinely helpful to observe colleagues in action. I continue to learn from others when they have different ways of navigating the system. This mutual exchange is crucial.” (Senior nurse)* |
|  | *“Much of it comes down to learning by doing. Colleagues often ask, “How do you do it? Do you have any advice?” Or you simply watch them and pick up on their efficient techniques. (Resident physician)* |
| Shortage of time for training | *“In my case, it felt like I was thrown into the deep end. Another resident physician quickly showed me some fundamentals, but my first day was definitely challenging. I wasn’t familiar with the process of ordering X-rays or lab tests, mainly because everything is conducted through the clinical information system I didn’t know.” (Resident physician)* |
|  | *“The challenging aspect is incorporating training sessions into our daily routines. Most people have packed schedules and struggle to find time for training. […] You could also attribute this to our leaders, as they often don’t allocate time for such training. Consequently, for many, it becomes a matter of learning by doing. Nevertheless, trainings are available and is actively promoted by the hospital to ensure everyone receives adequate training.” (Senior physician)* |
|  | *“However, if you’re working in the Emergency Department and are unfamiliar with their information system because it’s not your regular workplace, it can be quite challenging. You find yourself using software while caring for patients without truly understanding how that software functions.” (Resident physician)* |
| Desire for more training and effective knowledge sharing | *“However, there is no well-organized summary of knowledge available. You often come across individuals who have discovered something new on their own.” (Senior nurse)* |
|  | *“It’s unfortunate that there isn’t a standardized resource where all the program details are comprehensively explained, either in written or digital form.” (Staff nurse)* |
|  | *“In general, I’m in favor of digitization, but it needs to be well-established, thoroughly tested, and, most importantly, the team needs to be extensively trained, otherwise, it may result in more drawbacks than benefits.” (Senior nurse)* |

**Table S9.** Commonly reported factors on digital transformation and change (number of study participants).

| **IT Support model** | **Gaps and limitations** | **Innovation roadblocks** | **Opportunities** |
| --- | --- | --- | --- |
|  |  |  |  |
| Insufficient IT support, not tailored to needs (9) | Only limited use made of available technical possibilities (28) | Monetary reasons (15) | New clinical information system or parts of it in planning (22) |
| Internal IT department (8) | Desire for more digitalization and improvement (19) | Regulations and legal reasons (14) | Envisioning future tools (15) |
| Key-user as technical support (8) | Not Knowing all tool functions (12) | Unfavorable organizational structures (7) | HCP is involved in IT projects (11) |
| Technical support outside office hours (8) | No unified EHR available (8) | IT resources limited (2) | Vast opportunities and improvements (9) |
| Challenges for IT department (6) | Not knowing which tools are available (7) | Medical personnel and attitudes (2) | Digital might/should help to increase time with patient (4) |

**Table S10.** Supporting quotes on factors on digital transformation and change.

| **Topic** | **Reported quote** (role of study participant) |
| --- | --- |
|  |  |
| Change resistance | *However, the most resilient entity is the human being when it comes to resistance to change. So, yes, I always find that intriguing. Even when you believe you have a superior tool that genuinely streamlines your workflow or offers enhanced functionality, it remains challenging to persuade individuals in the healthcare field that it’s actually a positive change” (Senior physician)* |
|  | *Flexibility can be lacking at times, and resistance from people is natural because, ultimately, humans are just that - human. The willingness to embrace change has consistently been a challenge. However, we also observe this in leadership, and we have the ability to overcome these obstacles and continue our personal development.” (Senior nurse)* |
| Dynamics of IT provider and end-users | *“In that case, it was the manufacturer that provided the clinical information system, that showed no interest in creating a sufficient interface at all. This lack of interest posed a significant obstacle for a smooth user experience.” (Senior nurse)* |
|  | *“It’s often evident that certain criteria of end-user aren’t considered by those who create these programs.” (Senior physician)* |
|  | *“Certainly, there have been tools I’ve encountered or had to use that weren’t very intuitively designed or may have been developed by a team less familiar with the user’s perspective.” (Resident physician)* |
| Challenges in IT maintenance, support, and communication gaps | *“The system update wasn’t communicated beforehand. The idea that there’s something new implemented doesn’t even cross your mind when you’re focused on a sedated and ventilated patient. […] Suddenly, the buttons were in different places.” (Resident physician)* |
|  | *“There’s always a maintenance window on Thursday evenings, and we are sometimes affected by it. When the clinical information system is impacted, it disrupts our workflow significantly. For us, that’s quite a disaster. We are highly dependent on it.” (Senior nurse)* |
|  | *"The IT support team is putting in a lot of effort. I believe they too get frustrated by these disruptions. […] The team does its utmost. We report issues daily, including malfunction reports from this morning, for instance.” (Senior nurse)* |
| Attitude towards and understanding of digitalization | *“A physician who isn’t interested in or engaging with digital tools or AI hasn’t yet recognized the changing times.” (Senior physician)* |
|  | *“Because, in my opinion, simply digitizing what was previously in analog form doesn’t necessarily create added value. So, digitalization should somehow generate additional value.” (Senior nurse)* |
|  | *“When you ask people what digitalization means, they might say it’s taking a form that’s been around for 20 years, making a scan of it, and storing it electronically. That’s people’s understanding of digitalization, but it’s not entirely accurate. However, that’s where we are currently, moving from paper to digital, scanning, and calling it digitalization.” (Senior physician)* |
| Digitalization gap in healthcare | *“Unfortunately, we still have a long way to go when you consider the technological capabilities that are available”. (Senior physician)* |
|  | *“In healthcare, we are still lagging behind by several years compared to other industries.” (Senior physician)* |
|  | *“I find it very fascinating how quickly advancements happen in other fields, and how sluggish it is in medicine." (Resident physician)* |
| Challenges in e-health applications and electronic patient dossier | *“The introduction of the electronic patient dossier in Switzerland, which was aimed at achieving specific goals, has turned out to be quite a disaster. It seems we are far from where we wanted to be.” (Senior nurse)* |
|  | *“The electronic patient dossier is a significant topic, mandated by the federal government, but unfortunately, it has not been successfully implemented anywhere yet.” (Senior physician)* |
|  | *"E-health, particularly the patient dossier, has been somewhat of a tragedy in my opinion. It lacks structured information, especially regarding medications. While there is slow progress, it’s happening at a snail’s pace.” (Senior physician)* |
| End-users feel neglected in tool decision making | *"It appears that informatics projects are consistently challenging here. They tend to be extensive undertakings with high costs and involvement from numerous individuals, attempting to bundle everything in one solution.” (Senior nurse)* |
|  | *“In nursing, there are ongoing needs for development and improvements. However, the standard response you often hear is, “There’s no budget for that.” When it comes to digital initiatives, I need to submit project applications, secure project funds, and deal with the limitation of IT time resources.” (Senior nurse)* |
| Workarounds and development of own customized solutions | *“We also worked a lot on our own tools and started to develop software ourselves.” (Senior physician)* |
|  | *“We create what we need ourselves, bearing the cost. This way, we obtain tailor-made solutions that match our workflow, eliminating the need for redundant data entry.” (Senior physician)* |
| Future tools and potentials for improvement | *“There is a lot of room for optimization. For example, every patient receives a wristband with a scannable code. This code could serve various purposes, like medication administration. Digitalization offers numerous opportunities with significant potential.” (Senior nurse)* |
|  | *“What we are interested in is whether patients experience complications or have fatal outcomes related to specific diagnoses or other factors like gender, age, labs, and so on.” (Senior physician)* |
|  | *“One feature I would still like to have, is the ability to input and visualize data like blood pressure and blood sugar by looking at the patient’s data.” (Senior physician)* |
|  | *“Additionally, it would be beneficial to incorporate more visual elements into the patient’s room. I work with severely ill patients who may struggle to understand medical concepts. I rely heavily on visual aids, and I believe it would be beneficial if we could quickly show patients these visuals. For instance, I’m thinking of the use of 3D glasses for patients to help them better understand complex medical procedures, such as vascular systems. I find this idea incredibly exciting for better patient care.” (Senior nurse)* |
